# Influence of Hydroxyl Functional Group on the Structure and Stability of Xanthone: A Computational Approach

**DOI:** 10.3390/molecules23112962

**Published:** 2018-11-13

**Authors:** Vera L. S. Freitas, Maria D. M. C. Ribeiro da Silva

**Affiliations:** Centro de Investigação em Química da Universidade do Porto (CIQUP), Department of Chemistry and Biochemistry, Faculty of Sciences, University of Porto, Rua do Campo Alegre, P-4169-007 Porto, Portugal; mdsilva@fc.up.pt

**Keywords:** thermodynamic properties, gas-phase enthalpy of formation, tautomers, conformers, intramolecular hydrogen bond, monooxygenated xanthones

## Abstract

The present work addresses computational research focused on the energetic and structural properties of four isomers monohydroxyxanthone, using the G3(MP2)//B3LYP method, in order to evaluate the influence of the hydroxyl (—OH moiety) functional group on the xanthone molecule. The combination of these computational results with previous experimental data of these compounds enabled the determination of their enthalpies, entropies and Gibbs energies of formation, in the gaseous phase, and consequently to infer about the relative thermodynamic stability of the four isomers. Other issues were also addressed for the hydroxyxanthone isomers, namely the conformational and the tautomeric equilibrium analysis of the optimized molecular structures, the frontier orbitals, and the electrostatic potential energy maps. Complementarily, an energetic study of the intramolecular O—H⋯O hydrogen bond for 1-hydroxanthone was also performed.

## 1. Introduction

After the scientific community realized the potentialities of the biological and pharmacological properties of some xanthone derivatives and, the interest for other related compounds with a superior performance has gradually risen [1,2,3,4]. As the chemical nature and the position of the different substituents in the xanthone structure determine the aforementioned properties, several studies have been prompted to obtain new drug candidates, starting with natural products (higher plants, fungi and lichens) or using the organic synthesis.

A literature survey showed the scarcity of thermodynamic data for heteropolycyclic compounds that motivated our involvement in a systematic study of compounds structurally formed by two benzene rings fused to a pentagonal or hexagonal central ring, containing oxygen, sulfur or nitrogen as heteroatoms [5,6,7,8,9,10,11,12,13,14,15]. This work has been developed in a “duo dynamic” between the experimental and computational studies of those species, with the main goal of determining their thermodynamic properties, among others. Therefore, it has been possible to contribute with benchmark data for an appropriate database of thermodynamic parameters for these compounds, to support the development of computational studies for other compounds whose experimental study is not possible. Furthermore, the good agreement between the experimental and computational values obtained for the gas-phase enthalpy of formation enabled the validation of the computational procedure used to estimate this parameter for compounds of the same class.

The xanthone moiety has the potential to bind to a variety of targets, as well as to the hydroxyl functional group. Therefore, knowledge about the influence of this functional group in the xanthone structure and stability is important to understand more about these molecules—hydroxyxanthones—and to use this information in the drug design of new derivatives.

Hydroxyxanthones are abundantly found in many plants [16,17] and are reported to have a great deal of biological activities including vasorelaxing, antimalarial and antioxidative effects [18,19]. In particular, being a rich source of natural hydroxyxanthones, mangosteen (Garcinia mangostana) has long been used to treat diarrheal illness in traditional medicine, and more recently is considered a natural chemopreventive agent [20].

**Figure 1:**
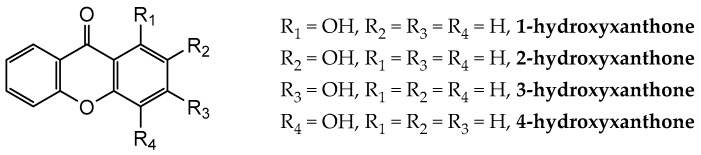
General molecular formulae of the monohydroxyxanthones studied in this work: 1-hydroxyxanthone (1OHXT), 2-hydroxyxanthone (2OHXT), 3-hydroxyxanthone (3OHXT), and 4-hydroxyxanthone (4OHXT).

In the present work, a computational study of the structural and thermochemical properties of four monohydroxyxanthones (1-hydroxyxanthone, 1OHXT, 2-hydroxyxanthone, 2OHXT, 3-hydroxyxanthone, 3OHXT, and 4-hydroxyxanthone, 4OHXT), with general molecular formula in Figure 1, has been carried out, through the use of the G3(MP2)//B3LYP composite method [21]. The development of the experimental work for these compounds was not performed due to the non-availability of samples in a large enough amount and purity.

The computational work focused on the conformational and tautomeric equilibrium analysis of the optimized molecular structures of the monohydroxyxanthone isomers and on the calculation of their standard (*p*° = 0.1 MPa) molar enthalpies, entropies and Gibbs energies of formation. The frontier molecular orbitals and the electrostatic potential energy surfaces are also addressed, together with the energy of the intramolecular hydrogen bond present in the 1-hydroxyxanthone isomer.

## 2. Results and Discussion

### 2.1. Conformational and Tautomeric Equilibrium Analysis

The molecular geometries of the monohydroxyxanthone isomers were optimized with the hybrid B3LYP method using the 6-31G(*d*) basis set—the first step from G3(MP2)//B3LYP composite method [21]. Each monohydroxyxanthone has at least two structural conformations owing to the possible arrangements of the hydroxyl substituent. The conformational composition, χ_i_, of each monohydroxyxanthone was calculated assuming a Boltzmann distribution of the *n* possible conformers.

The optimized geometries of the minima conformers in the potential energy surface, and the corresponding conformational composition, χ_i_ are presented in Table 1. Details of the conformational analysis performed are given in Supplementary Materials (Table S1). It should be noted that for 1-hydroxyxanthone the predominant conformation, with χ_i_ equal to 1, corresponds to the conformer I, where the hydrogen atom of the hydroxyl group is oriented towards the carbonyl oxygen, enabling the formation of an intramolecular hydrogen bond. For each one of the isomers the hydroxyl substituent is coplanar with the xanthone aromatic ring.

In previous reported works, for systems as xanthene/xanthone [6,8], it was verified that when the hybridization of the carbon of the central ring of xanthene changes from *sp*^3^ to *sp*^2^, as the result of the shift of a methylene group by a carbonyl group, the molecule changes from a folded structure to a planar one. This has been also evidenced for the following systems: thioxanthene/thioxanthone [7,9], 9,10-dihydroanthracene/anthrone [8], and 9,10-dihydroacridine/acridone [15,22].

The gas phase tautomeric keto-enol equilibrium of the monohydroxyxanthone isomers were also analysed using the Boltzmann’s distribution. The tautomeric fractions, χ, were calculated according the Equations (1) and (2), where $x_{\mathrm{keto}}$ and $x_{\mathrm{enol}}$ corresponds to the fractions of the keto and enol forms, respectively, and $\Delta_{f}G_{m}^{o}\left(g\right)$ is the gas-phase standard molar Gibbs energy of formation, *R* is the gas constant (*R* = 8.3144598 J K^−1^ mol^−1^) [23], and *T* is the reference temperature (298.15 K).

(1) $$x_{\mathrm{keto}}=\frac{e^{-[\Delta_{f}G_{m}^{o}\left(g\right)/RT]}}{1+e^{-[\Delta_{f}G_{m}^{o}\left(g\right)/RT]}}$$

(2) $$x_{\mathrm{enol}}=1-x_{\mathrm{keto}}$$

The results of these calculations presented in Table S2 of Supplementary Materials are unanimous: The tautomeric keto-enol equilibrium favours only the formation of the keto form for all the monohydroxanthones (the fraction $x_{\mathrm{keto}}$ is equal to 1).

### 2.2. Electrostatic Potential Energy Maps and Frontier Orbitals (HOMO and LUMO)

**Figure 2:**
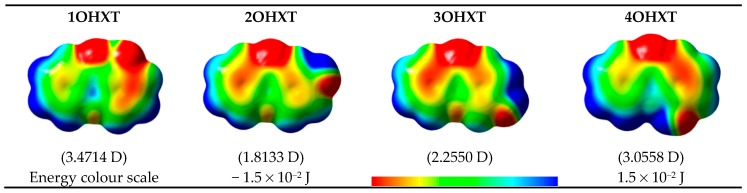
Electrostatic potential energy surfaces mapped onto an electron density isosurface (isovalue for spin density of 0.0004 *e*·*a*_0_^−3^; where *a*_0_ is the Bohr radius and corresponds to 5.2917721067 × 10^−11^ m [28] and *e* is the electron charge, 1.6021766208 × 10^−19^ C [28]), and dipole moment values for the global minimum conformer of each monohydroxyxanthone isomer.

The electrostatic potential energy map (EPEM) for each global minimum conformer of the monohydroxyxanthone was obtained from the Natural Bond Orbitals (NBO) calculations, performed using NBO 3.1 program [24], as implemented in the Gaussian 03 package [25], at the B3LYP/6-31G(*d*,*p*) level of theory [26,27]. These graphical models presented in Figure 2 provide information about the overall charge distribution of these molecules. The conventional choice for the colour map is the visible spectrum, representing the different intensities of the electrostatic potential energy values, i.e., red for the lowest electrostatic potential energy value (higher charge density), blue for the highest electrostatic potential energy value (lower charge density), and green for the electrostatic potential energy values near zero.

The carbonyl functional group is an electron-withdrawing by induction and resonance while the hydroxyl group is an electron-withdrawing by induction and electron-donating by resonance. The several positions of the hydroxyl substituent on xanthone originate different distributions in the electron density of the molecules, resulting also in different dipole moments, as can be seen in Figure 2.

In these graphical models, the regions that can be subject to attack by electrophiles and nucleophiles, are signed by the red and blue colours, respectively.

Views of the highest-occupied molecular orbitals (HOMO) and the lowest-unoccupied molecular orbitals (LUMO) for each global minimum conformer of the monohydroxyxanthones (obtained from natural bond orbital analyses performed with Gaussian NBO Version 3.1 [24]) are presented in Table 2, jointly with the corresponding energy gap, *E*_GAP_, calculated from the difference between the two, *E*_LUMO_ and *E*_HOMO_.

According to the NBO analysis, the electronic transitions from the HOMO (bonding oxygen lone pair orbital of the oxygen of the carbonyl group, $n_{O}^{\pi }$) to LUMO (antibonding C—Cπ bond involving the carbons of the benzene rings, $\pi_{C—C}^{*}$) for 2OHXT, 3OHXT, and 4OHXT isomers are of the type $n_{O}^{\pi }\to \pi_{C—C}^{*}$, with energy gaps between 7.04 and 7.18 eV. In the case of the 1OHXT isomer, the electronic transition from the HOMO (bonding C—Cπ bond involving the carbons of the benzene rings, $\pi_{C—C}^{}$) to LUMO (antibonding C = O π bond orbital of the carbonyl group, $\pi_{C=O}^{*}$) is of the type $\pi_{C—C}^{}\to \pi_{C=O}^{*}$, with a lower *E*_GAP_, suggesting a higher chemical reactivity relative to the other three isomers.

Usually, molecules with a low frontier orbital gap are associated with high chemical reactivity and low kinetic stability [29,30].

### 2.3. Intramolecular O—H⋯O Hydrogen Bonding Energetics in the 1-Hydroxyxanthone

In the global minimum conformer obtained for 1-hydroxyxanthone (I, Table 1), the orientation of the hydrogen of the hydroxyl group towards the oxygen of the carbonyl group and their close distance (1.70 × 10^−10^ m) enable the formation of an intramolecular hydrogen bond, resulting in a closed ring formation. This interaction was confirmed with theoretical analysis of the electron density topology, christened as Atoms in Molecules theory, AIM theory [31], by Bader, as implemented in the AIM11 program (Version 13.05.06) [32]. This analysis revealed a bond critical point between the mentioned interaction, with an electron density of *ρ* = 0.032 and a Laplacian of the electron density, $\nabla^{2}\rho$ = 0.21, and also the formation of a ring critical point due to this interaction.

According the Second-Order Perturbative theory analysis of Fock Matrix, in NBO basis [24], this intramolecular hydrogen bond is conventionally interpreted by the interaction between the “filled” Lewis-type NBO (donor NBO, i) and the “empty” non-Lewis NBO (acceptor NBO, j), as well as by the estimation of the stabilization energy associated with delocatization, $\Delta E_{\mathrm{ij}}^{2}$. The result of NBO analysis on the molecular system shows that the stabilization energy associated with the interaction between the oxygen lone pair ($n_{O}^{\pi }$) and the antibonding bond hydrogen-oxygen, $\sigma_{\mathrm{OH}}^{*}$, is 95.4 kJ mol^−1^.

To evaluate the energy of the intramolecular hydrogen bond, $E_{\mathrm{IMHB}}$, present in the 1OHXT isomer, we used the energies of the *cis* and *trans* conformers (both minima in the potential energy surface) obtained by the G3(MP2)//B3LYP method; their corresponding values are shown in Table 3. The energy difference between the *cis* and *trans* conformers of 1OHXT (I and II conformers represented in Table 1), rises to a value of $E_{\mathrm{IMHB}}$ = 49.4 kJ mol^−1^. This value resembles the value obtained previously in other studies for the same type of interaction [33]. According the X-ray diffraction experiments performed by Corrêa et al [34], this intramolecular hydrogen bond exists in the crystal state. In the same study, it was noticed that the hydroxyl group is not involved in any strong intermolecular hydrogen bonding, albeit hydroxyl groups can act as a strong proton acceptor and donor.

Qu and co-workers [35] performed a DFT computational study about the effect of hydroxyl groups on the stability and thermodynamic properties of polyhydroxylated xanthones. In that study the intramolecular hydrogen bond energy present in the 1OHXT, 52 kJ mol^−1^, was calculated from the difference between the Gibbs energy of formation of the *cis* and of the *trans* conformers. Using the data provided in Table S1 of Supplementary Materials, it is possible to carry out the same calculation, resulting in a close value, 46.4 kJ⋅mol^−1^. Comparing this last result with the one listed in Table 3, the low effect of the entropic contribution to the bond is confirmed.

### 2.4. Estimation of the Gas-Phase Standard Molar Enthalpies, Entropies and Gibbs Energies of Formation

The reliability of selected gas-phase enthalpies of formation is powerfully influenced by the computational method and a suitable set of chemical reactions. For polycyclic aromatic compounds such as the ones studied in this work, the isodesmic and homodesmotic reactions appear to be most appropriate [5,6,7,8,9,10,11,12,13,14,15]. These theoretical chemical reactions rely upon the similarity of the bonding environment of the reactants and products and lead to cancellation of systematic errors. The disadvantage of these reactions is the requirement of the knowledge of the experimental gas-phase enthalpies of formation of all the auxiliary molecules used. The availability of experimental data can limit the number of possibilities reactions. However, as previously mentioned, this study appears in the follow-up of a systematic experimental/computational study of heteropolycyclic compounds, therefore the number of available reliable experimental values allowed the use of 19 gas-phase hypothetical reactions, reported in Table 4. To avoid the use of any inaccurate experimental enthalpies of formation, reactions involving different auxiliary species were purposed.

The absolute standard enthalpies, $H_{298.15K}^{o}$, of all the species figured in the hypothetical gas-phase reactions were obtained computationally from the G3(MP2)//B3LYP method [21]. These data were used to calculate the gas-phase standard molar enthalpy of each working reaction, $\Delta_{R}H_{m}^{o}$, at *T* = 298.15 K, taking into account Equation (3). Thereupon, the rearrangement of Equation (4) and the knowledge of the experimental standard molar gas-phase enthalpies of formation of all the auxiliary species used enabled the calculation of the gas-phase enthalpy of formation for each monohydroxyxanthone. The G3(MP2)//B3LYP absolute enthalpies, $H_{298.15K}^{o}$, and the experimental enthalpies of formation in the gas phase, $\Delta_{f}H_{m}^{o}\left(g\right)$, of the molecular species used [8,9,15,22,36,37,38,39,40,41,42,43] are given in Table S3 of Supplementary Materials.

(3) $$\Delta_{R}H_{m}^{o}=\sum H_{298.15K}^{o}\left(\mathrm{products}\right)-\sum H_{298.15K}^{o}\left(\mathrm{reagents}\right)$$

(4) $$\Delta_{R}H_{m}^{o}=\sum \Delta_{f}H_{m}^{o}\left(\mathrm{products}\right)-\sum \Delta_{f}H_{m}^{o}\left(\mathrm{reagents}\right)$$

In Table 5, the estimated values for gas-phase enthalpies of formation for each of the monohydroxyxanthone conformers are reported, being obtained from the hypothetical reactions presented in Table 4. The final value of the gas-phase enthalpies of formation for each monohydroxyxanthone isomer was adjusted considering the contributions of each conformer (Table 5).

The standard gas-phase molar entropies of formation, $\Delta_{f}S_{m}^{o}\left(g\right)$, reported in Table 6, were calculated from the values of the standard absolute entropies at *T* = 298.15 K, $S_{298.15}^{o}\left(g\right)$, obtained by the G3(MP2)//B3LYP method. The vibrational frequencies were scaled by a factor of 1.0029 [44] (Table S1 in Supplementary Materials), and the following reference entropies were $\mathrm{used}:\;S_{m}^{o}$ (C, graphite) = 5.740 J K^−1^ mol^−1^, $S_{m}^{o}$(H_2_,g) = 130.680 J K^−1^ mol^−1^,and $S_{m}^{o}$(O_2_,g) = 205.147 J K^−1^ mol^−1^ [45]. The Gibbs energy, $\Delta_{f}G_{m}^{o}\left(g\right)$ at *T* = 298.15 K was calculated from from $\Delta_{f}G_{m}^{o}\left(g\right)=\Delta_{f}H_{m}^{o}\left(g\right)-T\Delta_{f}S_{m}^{o}(g)$.

Comparing the values of the gas-phase Gibbs energy of formation obtained for the monohydroxyxanthones, it is possible to notice that 1OHXT is the thermodynamically more stable isomer. This stabilization is due to the presence of the intramolecular hydrogen bond.

Qu and co-workers [35] also determined the enthalpies of formation and the Gibbs energies of formation of several polyhydroxylated xanthones using a DFT method (B3LYP/6-311G**) and some isodesmic reactions. Those results differ 13 to 20 kJ mol^−1^ from the ones obtained in our study (Table 6). We feel confident with our results, since the composite method applied in the development of the present study has been already tested for similar heteropolycyclic molecules, with the inherent validation of the methodology used [5,6,7,8,9,10,11,12,13,14,15].

## 3. Computational Method

Molecular calculations concerned with this work were performed with the Gaussian-03 software package [25] using the composite method G3(MP2)//B3LYP [21], a variation of Gaussian-3 (G3) theory [46].

The natural bonding orbital (NBO) analyses [24] were performed using NBO Version 3.1 program as implemented in the Gaussian-03 software package at the B3LYP/6-31G(*d*,*p*) level [26].

The topological properties of the electron density at the selected bond critical points (intramolecular hydrogen bonds) have been evaluated using the theory of atoms in molecules (AIM) [31] as implemented in the AIM11 program (version 13.11.04) [32].

## 4. Conclusions

The present computational study allowed the obtention of new and important thermodynamic parameters that characterize the gas-phase chemistry of the monohydroxyxanthone isomers.

From structural analysis, it was found that each monohydroxyxanthone has at least two minima conformers in the potential energy, whose composition was calculated using the Boltzmann distribution. The tautomeric keto-enol equilibrium was also analysed, demonstrating that the form keto is the predominant form.

From this set of four isomers, 1OHXT is the thermodynamically most stable isomer, essentially due to the presence of an intramolecular O—H⋯O hydrogen bond, whose energy was calculated as $E_{\mathrm{IMHB}}$ = 49.4 kJ mol^−1^. However, it should be highlighted that the isomer 1OHXT also presents the lowest frontier orbital gap and this may be related with a lower kinetic stability.

## Figures and Tables

**Table 1:** Conformational composition, χ_i_, for most stable molecular geometries obtained by G3(MP2)//B3LYP composite method for monohydroxyxanthone isomers (1OHXT, 2OHXT, 3OHXT, and 4OHXT) *^a^*.

	I	II
1OHXT	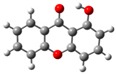	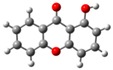
χ_i_	1.000	0
2OHXT	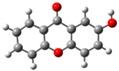	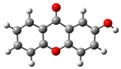
χ_i_	0.834	0.166
3OHXT	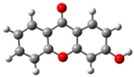	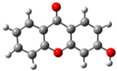
χ_i_	0.558	0.442
4OHXT	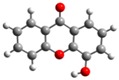	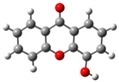
χ_i_	0.991	0.009

*^a^* Spheres colour code: grey, C; red, O; white, H.

**Table 2:** HOMO and LUMO maps (isovalue for molecular orbitals of 0.06 *e*⋅*a*_0_^−3^, where *a*_0_ is the Bohr radius) with the correspondent energies values, *E*_HOMO_ and *E*_LUMO_, and the calculated energy gap, *E*_GAP_. 1 eV corresponds to 1.6021766208 × 10^−19^ J [28] *^a^*.

	1OHXT	2OHXT	3OHXT	4OHXT
HOMO	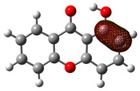	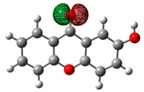	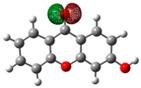	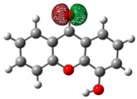
*E*_HOMO_/eV	−6.91	−6.65	−6.52	−6.72
LUMO	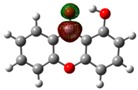	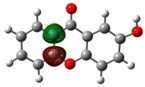	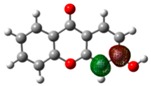	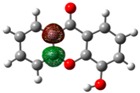
*E*_LUMO_/eV	−0.05	0.53	0.52	0.37
*E*_GAP_/eV	6.85	7.18	7.04	7.09

*^a^* Spheres colour code: grey, C; red, O; white, H.

**Table 3:** Energies values for 1-hydroxyxanthone conformers *cis* and *trans*, at *T* = 298.15 K, $E_{298.15K}^{o}$, obtained from G3(MP2)//B3LYP composite method for the calculation of the intramolecular hydrogen bond energy, $E_{\mathrm{IMHB}}$. 1 a. u. (Hartree) corresponds to 2625.50 kJ mol^−1^ ^*a*^.

Conformer *cis*	$E_{298.15K}^{o}/a.\;u.$	Conformer *trans*	$E_{298.15K}^{o}/a.\;u.$	$E_{\mathrm{IMHB}}$/kJ·mol^−1^
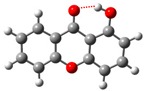	−724.834474	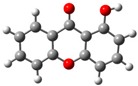	−724.815651	49.4

*^a^* Spheres colour code: grey, C; red, O; white, H.

**Table 4:** Hypothetical gas-phase reactions for the theoretical study of monohydroxyxanthones.

Reaction		Equations No.
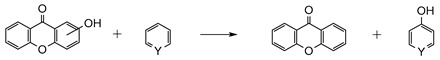	Y = CH	R1
	Y = N	R2
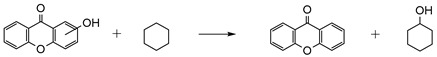		R3
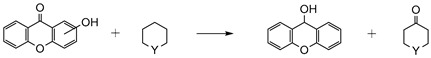	Y = CH_2_	R4
	Y = O	R5
	Y = S	R6
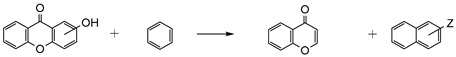	Z = 1-OH	R7
	Z = 2-OH	R8
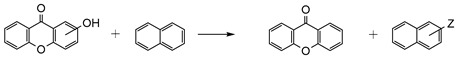	Z = 1-OH	R9
	Z = 2-OH	R10
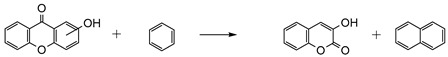		R11
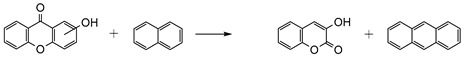		R12
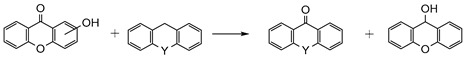	Y = CH_2_	R13
	Y = O	R14
	Y = S	R15
	Y = NH	R16
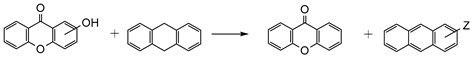	Z = 1-OH	R17
	Z = 2-OH	R18
	Z = 9-OH	R19

**Table 5:** Estimated standard gas-phase molar enthalpies of formation, $\Delta_{f}H_{m}^{o}\left(g\right)$, at *T* = 298.15 K for the monohydroxyxanthone conformers derived from hypothetical gas-phase reactions.

	1OHXT	2OHXT		3OHXT		4OHXT	
Conformer	I	I	II	I	II	I	II
**Equation No.**	$\Delta_{f}H_{m}^{o}(g)$/kJ mol^−1^						
**R1**	−302.51	−271.02	−266.60	−276.94	−276.44	−272.15	−260.33
**R2**	−297.90	−266.41	−261.99	−272.33	−271.83	−267.54	−255.72
**R3**	−301.77	−270.28	−265.87	−276.20	−275.71	−271.42	−259.59
**R4**	−298.20	−266.71	−262.29	−272.63	−272.13	−267.84	−256.01
**R5**	−303.61	−272.12	−267.70	−278.04	−277.54	−273.25	−261.43
**R6**	−300.76	−269.27	−264.85	−275.19	−274.69	−270.40	−258.58
**R7**	−297.85	−266.36	−261.94	−272.28	−271.78	−267.49	−255.67
**R8**	−299.24	−267.75	−263.33	−273.67	−273.17	−268.88	−257.06
**R9**	−303.50	−272.01	−267.59	−277.93	−277.43	−273.14	−261.32
**R10**	−304.89	−273.40	−268.98	−279.32	−278.82	−274.53	−262.71
**R11**	−304.91	−273.42	−269.00	−279.34	−278.84	−274.55	−262.73
**R12**	−307.42	−275.93	−271.51	−281.85	−281.35	−277.06	−265.24
**R13**	−302.20	−270.71	−266.29	−276.63	−276.13	−271.84	−260.02
**R14**	−296.25	−264.76	−260.34	−270.68	−270.18	−265.89	−254.07
**R15**	−305.62	−274.13	−269.71	−280.05	−279.55	−275.26	−263.43
**R16**	−299.62	−268.13	−263.71	−274.05	−273.55	−269.26	−257.44
**R17**	−301.90	−270.41	−265.99	−276.33	−275.84	−271.55	−259.72
**R18**	−301.34	−269.85	−265.43	−275.77	−275.27	−270.98	−259.16
**R19**	−299.49	−268.00	−263.58	−273.92	−273.42	−269.13	−257.31
Mean value *^a^*	−301.5 ± 2.9	−270.0 ± 2.9	−265.6 ± 2.9	−276.0 ± 2.9	−275.5 ± 2.9	−271.2 ± 2.9	−259.3 ± 2.9
X *^b^*	1.000	0.834	0.166	0.558	0.442	0.991	0.009
Final value *^c^*	−301.5 ± 2.9	−269.3 ± 2.9		−275.8 ± 2.9		−271.1 ± 2.9	

*^a^* The uncertainty assigned correspond to the expanded uncertainty determined from the estimated standard deviation of the mean for 19 reactions and the coverage factor *k* = 2.101 (0.95 level of confidence and 18 degrees of freedom); *^b^* Conformational composition; *^c^* Final values for the gas-phase enthalpies of formation considering the conformation composition.

**Table 6:** Estimated standard gas-phase molar enthalpies, entropies, and Gibbs energies of formation, respectively, $\Delta_{f}H_{m}^{o}\left(g\right),$ $\Delta_{f}S_{m}^{o}\left(g\right)$ $\Delta_{f}G_{m}^{o}\left(g\right)$ for the monohydroxyxanthone isomers. at *T* = 298.15 K.

Isomers	$\Delta_{f}H_{m}^{o}\left(g\right)$/kJ mol^−1^^*a*^	$\Delta_{f}S_{m}^{o}\left(g\right)$/J K^−1^ mol^−1^ ^*b*^	$\Delta_{f}G_{m}^{o}\left(g\right)$/kJ mol^−1^^*c*^
1OHXT	−301.5 ± 2.9	−475.8	−159.6
2OHXT	−269.3 ± 2.9	−466.8	−130.2
3OHXT	−275.8 ± 2.9	−467.1	−136.6
4OHXT	−271.1 ± 2.9	−467.5	−131.7

*^a^* Final value obtained in Table 5; *^b^* The calculation method of $\Delta_{f}S_{m}^{o}\left(g\right)$ is given in more detail in Supplementary Materials (Table S1); *^c^* Calculated from $\Delta_{f}G_{m}^{o}\left(g\right)=\Delta_{f}H_{m}^{o}\left(g\right)-T\Delta_{f}S_{m}^{o}(g)$.

## Supplementary Materials

The following are available online. Table S1. Absolute standard enthalpies and entropies obtained by G3(MP2)//B3LYP composite method for monohydroxyxanthone conformers, and the corresponding derived gas-phase standard molar enthalpies, entropies, and Gibbs energy of formation, at *T* = 298.15 K, together with the conformational composition. Table S2. Gas-phase absolute standard Gibbs energies, $G_{298.15K}^{o}$, obtained by G3(MP2)//B3LYP composite method for keto and enol forms of monohydroxyxanthones isomers, and the theoretically predicted gas-phase standard molar Gibbs energies, $\Delta_{r}G_{m}^{o}\left(g\right)$, for the keto-enol equilibrium, at *T* = 298.15 K, with the corresponding fractions (*x*) of the two tautomers. Table S3. G3(MP2)//B3LYP enthalpies with corresponding conformer composition, and experimental gas-phase standard molar enthalpies of formation, at *T* = 298.15 K, for monohydroxyxanthone isomers and for the auxiliary species.
