# Supplementary material for: Influence of Hydroxyl Functional Group on the Structure and Stability of Xanthone: A Computational Approach
Source: Molecules. 2018 Nov 13;23(11):2962. doi: 10.3390/molecules23112962 (PMC6280152; doi:10.3390/molecules23112962)
Supplement: Supplementary file 1 [file molecules-23-02962-s001.pdf]

Influence of hydroxyl functional group on the structure and stability of xanthone: a computational approach

Vera L. S. Freitas [1,*], Maria D. M. C. Ribeiro da Silva [1]

[1] Centro de Investigação em Química da Universidade do Porto (CIQUP), Department of Chemistry and Biochemistry, Faculty of Science, University of Porto, Rua do Campo Alegre, P-4169-007 Porto, Portugal; mdsilva@fc.up.pt (M.D.M.C.R.S.)

* Correspondence: vera.freitas@fc.up.pt; Tel.: +35-122-040-2538

Received: date; Accepted: date; Published: date

Appendix A

Table of Contents

		page
**Table A1.**	Absolute standard enthalpies, $H^{\circ}_{298.15\ \mathrm{K}}$, and entropies, $S^{\circ}_{298.15\ \mathrm{K}}$, obtained by G3(MP2)//B3LYP composite method for 1-hydroxyxanthone (1OHXT), 2-hydroxyxanthone (2OHXT), 3-hydroxyxanthone (3OHXT), and 4-hydroxyxanthone (4OHXT), conformers, and the corresponding derived gas-phase standard molar enthalpies, $\Delta_f H^{\circ}_m$(g), entropies, $\Delta_f S^{\circ}_m$(g), and Gibbs energy of formation, $\Delta_f G^{\circ}_m$(g), at T = 298.15 K, and the conformational composition, χ_I …….	A2
**Table A2.**	Gas-phase absolute standard Gibbs energies, $G^{\circ}_{298.15K}$, obtained by G3(MP2)//B3LYP composite method for keto and enol forms of monohydroxyxanthones isomers, and the theoretically predicted gas-phase standard molar Gibbs energies, $\Delta_r G^{\circ}_m$(g), for the keto-enol equilibrium, at T = 298.15 K, with the corresponding fractions (x) of the two tautomers ……………………………….	A4
**Table A3.**	G3(MP2)//B3LYP enthalpies, $H^{\circ}_{298.15K}$, with corresponding conformer composition (χi), and experimental gas-phase standard ($p°$ = 0.1 MPa) molar enthalpies of formation, $\Delta_f H^{\circ}_m$(g), at T = 298.15 K, for monohydroxyxanthone isomers and for the auxiliary species ………………………	A5

Acronims used throughout this supplementary data:
1OHXT for 1-hydroxyxanthone
2OHXT for 2-hydroxyxanthone
3OHXT for 3-hydroxyxanthone
4OHXT for 4-hydroxyxanthone

Standard thermodynamic property:
The standard state of a pure gas refers to its standard state is that of an ideal gas at p of 0.1 MPa (or, which is equivalent, that of a real gas at p = 0).
Standard states will be denoted by a superscript "o".

Table A1. Absolute standard enthalpies, $H^{\circ}_{298.15\ \mathrm{K}}$, and entropies, $S^{\circ}_{298.15\ \mathrm{K}}$, obtained by G3(MP2)//B3LYP composite method for 1-hydroxyxanthone (1OHXT), 2-hydroxyxanthone (2OHXT), 3-hydroxyxanthone (3OHXT), and 4-hydroxyxanthone (4OHXT), conformers, and the corresponding derived gas-phase standard molar enthalpies, $\Delta_f H^{\circ}_{m}(g)$, entropies, $\Delta_f S^{\circ}_{m}(g)$, and Gibbs energy of formation, $\Delta_f G^{\circ}_{m}(g)$, at T = 298.15 K, and the conformational composition, χ_i. 1 a. u. (Hartree) corresponds to 2625.50 kJ·mol⁻¹.

Specie	Conformation[a]	$H^{\circ}_{298.15K}$ [b] / a.u.	$\Delta_f H^{\circ}_{m}(g)$ [c] / kJ·mol⁻¹	$S^{\circ}_{298.15K}$ [d] / J·K⁻¹·mol⁻¹	$\Delta_f S^{\circ}_{m}(g)$ [e] / J·K⁻¹·mol⁻¹	$\Delta_f G^{\circ}_{m}(g)$ [f] / kJ·mol⁻¹	χ_i [g]
**1OHXT**	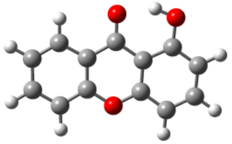	–724.833530	–301.5 ± 2.9	429.22	–475.8	–159.6	1.000
	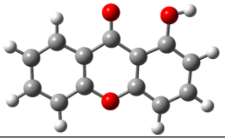	–724.814707	–252.1 ± 2.9	439.20	–465.9	–113.2	0
**2OHXT**	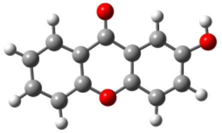	–724.821536	–270.0 ± 2.9	438.09	–467.0	–130.8	0.834
	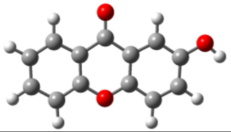	–724.819853	–265.6 ± 2.9	439.40	–465.7	–126.8	0.166
**3OHXT**	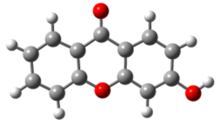	–724.823791	–276.0 ± 2.9	438.08	–467.0	–136.8	0.558
	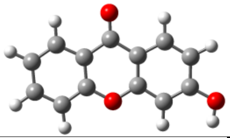	–724.823601	–275.5 ± 2.9	437.83	–467.20	–136.2	0.442
**4OHXT**	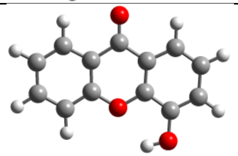	–724.821967	–271.2 ± 2.9	437.59	–467.7	–131.8	0.991
	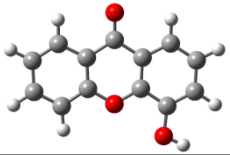	–724.817464	–259.3 ± 2.9	438.49	–466.6	–120.2	0.009

[a]Spheres color code: grey, C; red, O; white, H.
[b]Obtained from G3(MP2)//B3LYP method [1]
[c]Estimated from 19 working reactions presented on Table 1 of manuscript;
[d]Obtained from B3LYP/6-31G(d) method for a frequency factor scale of 1.0029 [2];
[e]Calculated from $\Delta_f S^\circ_m(g) = S^\circ_{298.15K}$ (conformer i) $- \sum S^\circ_{298.15K}$ (elements), considering the standard absolute entropy elements values, at 298.15 K, $S^\circ_{298.15K}\left(H_2, g\right)$ = 130.680 J·K^{-1}·mol^{-1}, $S^\circ_{298.15K}\left(C, \text{graphite}\right)$ = 5.740 J·K^{-1}·mol^{-1} and $S^\circ_{298.15K}\left(O_2, g\right)$ = 205.147 J·K^{-1}·mol^{-1} taken from ref. [3];
[f]Calculated from $\Delta_f G^\circ_m(g) = \Delta_f H^\circ_m(g) - T\Delta_f S^\circ_m(g)$;
[g] Calculated from $\chi_i = e^{-[\Delta_f G^\circ_m(g)/RT]}/\sum_i^n e^{-[\Delta_f G^\circ_m(g)/RT]}$.

Table A2. Gas-phase absolute standard Gibbs energies, $G^\circ_{298.15K}$, obtained by G3(MP2)//B3LYP composite method for keto and enol forms of monohydroxyxanthones isomers, and the theoretically predicted gas-phase standard molar Gibbs energies, $\Delta_r G^\circ_m(g)$, for the keto-enol equilibrium, at T = 298.15 K, with the corresponding fractions (x) of the two tautomers. 1 a. u. (Hartree) corresponds to 2625.50 kJ·mol^{-1}.[a]

	**1OHXT**		**2OHXT**	
	**keto form**	**enol form**	**keto form**	**enol form**
	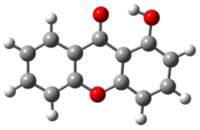	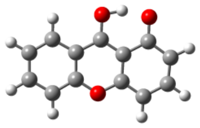	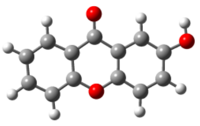	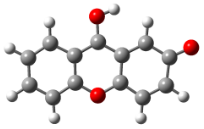
$G^\circ_{298.15K}$ /a. u.	–724.883106	–724.866965	–724.872146	–724.816688
$\Delta_r G^\circ_m(g)$ [b]/kJ·mol^{-1}	–42.4		–145.6	
fraction[c]	x_{keto} = 1.0	x_{enol} = 0	x_{keto} = 1.0	x_{enol} = 0
	**3OHXT**		**4OHXT**	
	**keto form**	**enol form**	**keto form**	**enol form**
	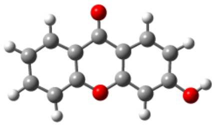	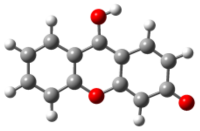	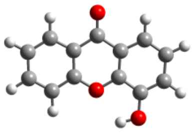	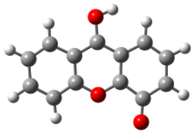
$G^\circ_{298.15K}$ /a. u.	–724.874401	–724.845105	–724.872519	–724.814434
$\Delta_r G^\circ_m(g)$ [b] / kJ·mol^{-1}	–76.9		–152.5	
fraction[c]	x_{keto} = 1.0	x_{enol} = 0	x_{keto} = 1.0	x_{enol} = 0

[a] Most stable conformation; Spheres color code: grey, C; red, O; and white, H.

[b] Calculated from $\Delta_r G^\circ_m(g) = G^\circ_{298.15\,K}(\text{keto}) - G^\circ_{298.15\,K}(\text{enol})$;

[c] Calculated according: $x_{keto} = \frac{e^{-[\Delta_f G^\circ_m(g)/RT]}}{1+e^{-[\Delta_f G^\circ_m(g)/RT]}}$ and $x_{enol} = 1 - x_{keto}$.

Table A3. G3(MP2)//B3LYP enthalpies, $H^{\circ}_{298.15\mathrm{K}}$, with corresponding conformer composition (χi), and experimental gas-phase standard ($p°$ = 0.1 MPa) molar enthalpies of formation, $\Delta_f H^{\circ}_m$(g), at T = 298.15 K, for monohydroxyxanthone isomers and for the auxiliary species. 1 a. u. (Hartree) corresponds to 2625.50 kJ·mol-1.

Compound	Molecular structure	$H^{\circ}_{298.15\mathrm{K}}$ / a. u. (χi)[a]	$\Delta_f H^{\circ}_m$(g) / kJ·mol-1
acridin-9(10*H*)-one	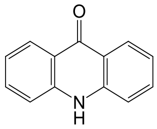	−629.817480	50.0 ± 5.0 [4]
anthracene	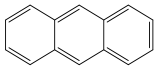	−538.606511	230.9 ± 2.2 [5]
1-anthrol	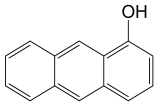	−613.758408 (**0.847**)<br>−613.756318 (**0.153**)	50.3 ± 5.3 [6]
2-anthrol	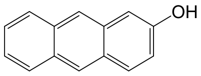	−613.758495 (**0.721**)<br>−613.757394 (**0.279**)	50.6 ± 5.3 [6]
9-anthrol	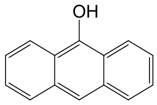	−613.756647	56.5 ± 5.3 [6]
anthrone	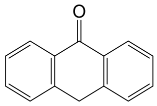	−613.762341	36.1 ± 3.2 [7]
benzene	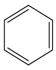	−231.835164	82.6 ± 0.7 [5]
4*H*-chromen-4-one	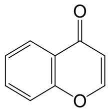	−496.270967	−126.1 ± 2.5 [8]
cyclohexane	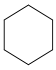	−235.407852	−123.3 ± 0.8 [5]
cyclohexanol	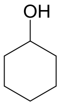	−310.557138 (**0.717**)<br>−310.556391 (**0.283**)	− 295.7 ± 1.2[a] [9,10]
cyclohexanone	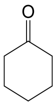	−309.367618	−226.1 ± 2.1 [5]
9,10-dihydroacridine	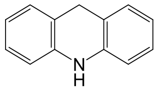	−555.840773	198.7 ± 4.4 [11]

…/…

Table A3. (Continuation)

Compound	Molecular structure	$H^{\circ}_{298.15K}$ / a. u. (χ_i)[a]	$\Delta_f H^{\circ}_m$(g) / kJ·mol^{-1}
9,10-dihydroanthracene	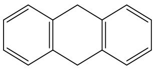	–539.796179	159.7 ± 4.3 [5]
3-hydroxycoumarin	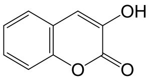	–571.443052	–367.7 ± 1.9 [12]
1-hydroxynaphthalene (or 1-naphthol)	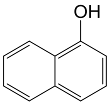	–460.375351	–30.4 ± 1.6 [13]
2-hydroxynaphthalene	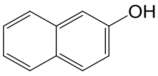	–460.375409	–29.9 ± 1.7 [13]
naphthalene	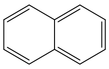	–385.223772	150.3 ± 1.4 [5]
phenol	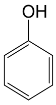	–306.986472	–96.4 ± 0.9 [5]
pyridine	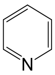	–247.873432	140.4 ± 0.7 [5]
pyridin-4-ol	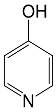	–323.027334	–40.8 ± 5.3 [14]
tetrahydro-2*H*-thiopyran	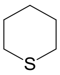	–593.928601	–63.5 ± 1.0 [5]
tetrahydro-2*H*-pyran	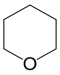	–271.303681	–223.8 ± 1.0 [5]
tetrahydro-4*H*-thiopyran-4-one	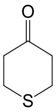	–667.886742	–164.6 ± 2.0 [15]
tetrahydro-4*H*-pyran-4-one	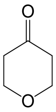	–345.262186	–328.7 ± 2.6 [16]
thioxanthene	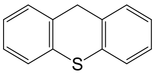	–898.319446	218.7 ± 4.2 [17]

…/…

Table A3. (Continuation)

Compound	Molecular structure	$H^{\circ}_{298.15K}$ / a. u. (χ_i)[a]	$\Delta_f H^{\circ}_m$(g) / kJ·mol^{-1}
thioxanthone	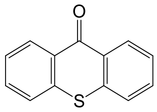	−972.285527	91.9 ± 2.4 [15]
xanthene	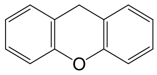	−575.697906	41.8 ± 3.5 [7]
xanthone	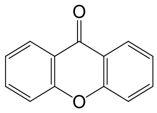	−649.670982	−94.0 ± 4.6 [16]
xanthydrol	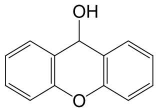	−650.845504	−121.2 ± 4.6 [18]

a Calculated from the standard molar enthalpy of formation in the liquid phase given in ref. [9] and from the standard molar enthalpy of vaporization given in ref. [10], at T = 298.15 K,

References

1. Baboul, A.G.; Curtiss, L.A.; Redfern, P.C.; Raghavachari, K. Gaussian-3 theory using density functional geometries and zero-point energies. *J. Chem. Phys.* **1999**, *110*, 7650-7657 [http://dx.doi.org/10.1063/1.478676]
2. Merrick, P.; Moran, D.; Radom, L. An evaluation of harmonic vibrational frequency scale factor, *J. Phys. Chem. A* 2007, 111 11683-11700 [http://dx.doi.org/ 10.1021/jp073974n]
3. Chase Jr.,M.W. Nist-Janaf Thermochemical Tables. *J Phys Chem Ref Data* **1998**, Monograph 9 (part I and II), 1-1951. Available online: https://janaf.nist.gov/ (accessed on 29 september 2018).
4. Freitas, V.L.S.; Ferreira, P.J.O., Ribeiro da Silva, M.D.M.C. Experimental and computational thermochemical studies of acridone and *N*-methylacridone. *J. Chem. Thermodyn.* **2018**, *118*, 115-126 [http://dx.doi.org/10.1016/j.jct.2017.11.002].
5. Pedley J.B. Thermochemical data and structures of organic compounds, Vol. 1; College Station, Thermodynamics Research Centre: Texas, USA, 1994, ISBN: 9781883400019.
6. Notario, R.; Roux, M.V.; Liebman, J.F. The energetics of the isomeric anthrols. *Mol. Phys.* **2004**, *102*, 623–625 [http://dx.doi.org/10.1080/00268970410001671549].
7. Freitas, V.L.S.; Gomes, J.R.B.; Ribeiro da Silva, M.D.M.C. Energetic effects of ether and ketone functional groups in 9,10-dihydroanthracene compound. *J. Chem. Thermodyn.* **2010**, *42*, 1248-1254. [http://dx.doi.org/10.1016/j.jct.2010.04.027].
8. Matos, M.A.R.; Sousa, C.C.S.; Miranda, M.S.; Morais, V.M.F.; Liebman, J.F. Energetics of coumarin and chromone. J. Phys. Chem. B 2009, 113, 11216-11221 [http://dx.doi.org/10.1021/jp9026942].
9. Kelley, K.K. Cyclohexanol and the third law of thermodynamics. *J. Am. Chem. Soc.* **1929**, *51*, 1400-1406 [http://dx.doi.org/10.1021/ja01380a014].
10. Steele, W.V.; Chirico, R.D.; Knipmeyer, S.E.; Nguyen, A.; Smith, N.K. Vapor Pressure, heat capacity, and density along the saturation line, measurements for cyclohexanol, 2-cyclohexen-1-one, 1,2-dichloropropane, 1,4-di-*tert*-butylbenzene, (±)-2-ethylhexanoic acid, 2-(methylamino)ethanol, perfluoro-*n*-heptane, and sulfolane. *J. Chem. Eng. Data* **1997**, *42*, 1021-1036 [http://dx.doi.org/ 10.1021/je9701036].
11. Freitas, V.L.S.; Gomes, J.R.B.; Liebman, J.F.; Ribeiro da Silva, M.D.M.C. Energetic and reactivity properties of 9,10-dihydroacridine and diphenylamine: a comparative overview. *J. Chem. Thermodyn.* **2017**, *11*, 5276-284 [http://dx.doi.org/10.1016/j.jct.2017.08.001].
12. Sousa, C.C.S.; Morais, V.M.F.; Matos, M.A.R. Energetics of the isomers: 3- and 4-hydroxycoumarin. *J. Chem. Thermodyn.* **2010**, *42*, 1372-1378 [http://dx.doi.org/10.1016/j.jct.2010.06.003].
13. Ribeiro da Silva, M.A.V.; Ribeiro da Silva, M.D.M.C.; Pilcher, G Enthalpies of combustion of 1-hydroxynaphthalene, 2-hydroxynaphthalene, and 1,2-, 1,3-, 1,4-, and 2,3-dihydroxynaphthalenes. *J. Chem. Thermodyn.* **1988**, *20*, 969-997 [http://dx.doi.org/10.1016/0021-9614(88)90225-X].
14. Ribeiro da Silva, M.A.V.; Matos, M.A.R.; Meng-Yan, Y.; Pilcher, G. Enthalpy of formation of 4-hydroxypyridine. *J. Chem. Thermodyn.* **1992**, *24*, 107-108 [http://dx.doi.org/ 10.1016/S0021-9614(05)80261-7].
15. Freitas, V.L.S.; Gomes, J.R.B.; Gales, L.; Damas, A.M.; Ribeiro da Silva, M.D.M.C. Experimental and computational studies on the structural and thermodynamic properties of two sulfur heterocyclic keto compounds. *J. Chem. Eng. Data* **2010**, *55*, 5009-5017 [http://dx.doi.org/10.1021/je100603q].
16. Freitas, V.L.S.; Gomes, J.R.B.; Ribeiro da Silva, M.D.M.C. Energetic studies of two oxygen heterocyclic compounds: xanthone and tetrahydro-γ-pyrone. *J. Therm. Anal. Calorim.* **2009**, *97*, 827-833. [http://dx.doi.org/10.1007/s10973-009-0233-y].
17. Freitas, V.L.S.; Monte, M.J.S.; Santos, L.M.N.B.F.; Gomes, J.R.B.; Ribeiro da Silva, M.D.M.C. Energetic studies and phase diagram of thioxanthene. *J. Phys. Chem. A* **2009**, *113*, 12988-12994. [http://dx.doi.org/10.1021/jp906413y].
18. Freitas, V.L.S.; Gomes, J.R.B.; Ribeiro da Silva, M.D.M.C. Experimental and computational thermochemical studies of 9-R-xanthene derivatives (R=OH, COOH, CONH2). *J. Chem. Thermodyn.* **2012**, *54*, 108-117. [http://dx.doi.org/10.1016/j.jct.2012.03.017].
